# Investigation of Frontal Lobe Activation with fNIRS and Systemic Changes During Video Gaming

**DOI:** 10.1007/978-1-4614-7411-1_13

**Published:** 2013-03-25

**Authors:** Ilias Tachtsidis, Antonis Papaioannou

**Affiliations:** Department of Medical Physics & Bioengineering, University College London, Malet Place Engineering Building, London, UK

**Keywords:** Video Gaming, Mean Blood Pressure, Game Task, Puzzle Game, Finapres Medical System

## Abstract

Frontal lobe activation caused by tasks such as videogames can be investigated using multichannel near-infrared spectroscopy (fNIRS), sometimes called optical topography. The aims of this study are to investigate the effects of video gaming (fighting and puzzle games) in the brain and the systemic physiology and to determine whether systemic responses during the gaming task are associated with the measurement of localised cerebral haemodynamic changes as measured by fNIRS. We used a continuous-wave 8-channel fNIRS system to measure the changes in concentration of oxy-haemoglobin (HbO_2_) and deoxy-haemoglobin (HHb) and changes in total haemoglobin (ΔtHb = ΔHbO_2_ + ΔHHb) over the frontal lobe in 30 healthy volunteers. The Portapres system was used to measure mean blood pressure (MBP) and heart rate (HR), and a laser Doppler was employed to measure the changes in scalp blood flow (or flux). Even though we observed significant changes in systemic variables during gaming, in particular in scalp flow, we also managed to see localised activation patterns over the frontal polar (FP1) region. However, in some channels over the frontal lobe, we also observed significant correlations between the HbO_2_ and systemic variables.

## Introduction

Multichannel functional near-infrared spectroscopy (fNIRS), or optical topography (OT), is often employed to detect brain functional activation. fNIRS measures the changes in brain tissue concentrations of oxy-haemoglobin (HbO_2_) and deoxy-haemoglobin (HHb) that occur secondary to the brain electrical activity changes due to the activation task. The fNIRS haemodynamic changes should occur at specific locations that overlay the cortical activated areas and should be closely coupled to the task-related timing periods. This assumes that the functional haemodynamic task-related changes are occurring on top of an unchanged global systemic and brain resting state. However, in certain functional experiments, these assumptions are not accurate and can lead to false positives in fNIRS [1]. We have previously used fNIRS to monitor the frontal and prefrontal cortex during anagram-solving tasks and observed significant task-related changes in mean blood pressure (MBP), heart rate (HR) and scalp blood flow (flux) that correlated with the fNIRS signals [2, 3]. In a recent study using multichannel fNIRS to produce maps of the haemodynamic response during anagram solving while simultaneously monitoring the systemic physiology, we observed a number of fNIRS channels to be highly correlated with activation-related systemic changes leading to false-positive cortical activation locations [1]. In our latest study utilising both fNIRS and functional magnetic resonance imaging (fMRI) and angiography during frontal activation tasks, we observed a significant correlation between the changes in HbO_2_ and the systemic activation response of the deep scalp veins [4].

Several earlier studies used fNIRS to investigate the effect of videogames over the frontal and prefrontal lobe [5, 6]; however, none of these studies investigated the systemic changes during this type of activation task. The main aims of this study are to determine whether there are significant systemic changes during video gaming and if these changes are significantly associated with the fNIRS haemodynamic measurements.

## Methods

We used two commercially available videogames for the Game Boy Advance SP (Nintendo Corp. Japan), a ‘fighting’ game (Final Fight One, Capcom) and a ‘puzzle’ game (Polarium Advance, Nintendo). The former is an arcade game where the player can choose a hero and fight against different enemies in order to complete specific missions. The latter is a very simple puzzle game, where the player has to flip black or white tiles on a square board in order to create horizontal rows of one colour and erase all the tiles in a single stroke to clear the board.

We studied two groups of healthy young volunteers, most of whom had some previous experience in video gaming. The first group (*n* = 17, mean age 24 years) did the puzzle game and the second group (*n* = 13, mean age 24 years) did the fighting game. These studies were approved by the Research Ethics Committee of UCL.

In order to become familiar with the experimental environment, the volunteers were given the game to practise for about 10 min. Following that, each subject sat in front of a desk on which a computer monitor was placed to alert the subjects via a visual stimulus when to rest and when to start playing the game. The protocol involved a single block of 5 min playing the game continuously (activation period) with a 2-min period of rest before and after the activation block.

**Fig. 13.1:**
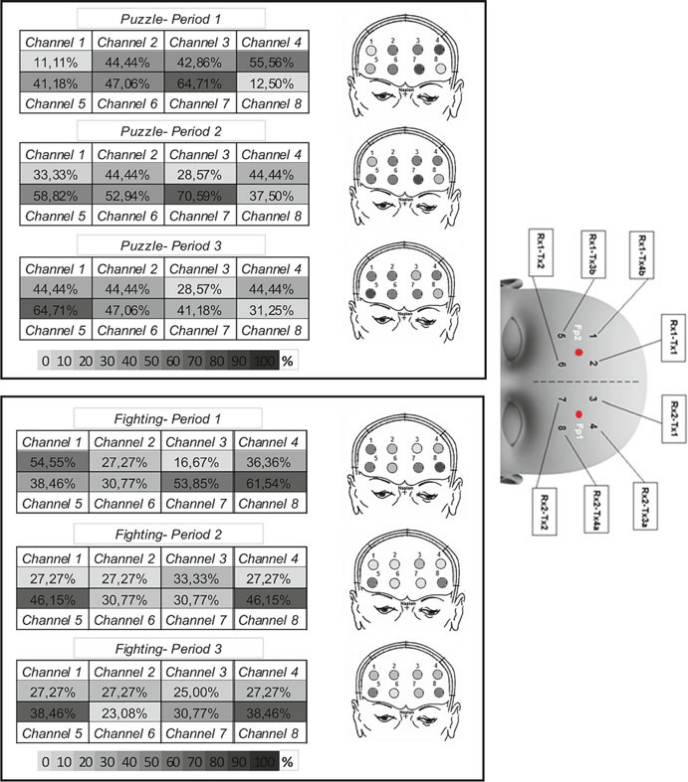
Group analysis shows the percentage of subjects that demonstrated activation in specific channels and epochs

A continuous-wave (CW) 8-channel fNIRS system, the Oxymon Mk III (Artinis Medical Systems BV, The Netherlands), was used. This system measures the changes in light attenuation at two wavelengths, 764 nm and 858 nm, and utilises the modified Beer-Lambert law with an age-dependent differential path length factor (DPF) to resolve the concentration changes in oxy (HbO_2_)- and deoxy (HHb)-haemoglobin and calculate the changes in total haemoglobin (tHb) which is the summation of ΔHbO_2_ and ΔHHb. The optode (source-detector fibre) configuration used in this study was the 8-channel split, which allows eight channel recordings with an inter-optode distance of 40 mm. The optode template was placed on the volunteer’s forehead, using the international 10/20 system of electrode placement [7], such that (i) FP1 region was covered between light-emitting fibres (Tx2, Tx4a) and light-receiving fibre (Rx2), corresponding to channels 7 and 8, and (ii) FP2 region was covered between light-emitting fibres (Tx2, Tx3b) and light-receiving fibre (Rx1), corresponding to channels 5 and 6 (Fig. 13.1).

The Portapres system (Finapres Medical Systems) was employed, with the inflatable cuff placed on the index finger of the left hand, in order to measure the mean blood pressure (MBP) and the heart rate (HR). The laser Doppler (Moor Instruments) was used to measure the scalp blood flow (flux) with the laser probe placed on the forehead.

In order to locate the activation channels, the activation period was split in three separate epochs each one having a duration of 10 s. The first epoch was immediately after the beginning of the stimuli (120–129 s); the second was in the middle of the activation period (270–279 s) and the third was at the end of the activation (411–420 s). For each epoch, we calculate a mean value for all measurements and subtracted that from a 10-s mean calculated at the beginning of the rest period (1–10 s). The difference was then compared to zero using a Student’s *t*-test to assess the significance (*p* ≤ 0.05). We then defined activation as a significant increase in HbO_2_, a significant decrease or no change in HHb and a significant increase in tHb [3]. Further, we estimated the correlation between the fNIRS and systemic signals to assess the relation of the brain haemodynamic and systemic signals. No correlation was defined as 0.25 > *r* > −0.25.

## Results

Figure 13.1 presents a summary of the percentage of subjects that demonstrated activation for each fNIRS channel. For the fighting game: in the first epoch 62 % of the subjects show activation over channel 8; in the second epoch, 46 % of the subjects show activation (equally) over both channel 5 and 8, while during the third epoch 39 % of the subjects show activation (equally) over both channels 5 and 8. For the puzzle game: in the first and second epochs, 65 % and 71 % of the subjects, respectively, demonstrated activation over channel 7, while in the third epoch, 65 % of the subjects demonstrated activation over channel 5 and only 31 % of the subjects show activation over channel 7.

Analysis of the systemic data showed that synchronous with the cerebral haemodynamic changes, significant systemic responses occurred as well. Individual analysis showed that during the fighting game, 77 % of the subjects showed a significant change in at least one of the systemic variables, with MBP and HR showing a consistent increase during the second and third epochs, while flux demonstrated an increase during all epochs. During the puzzle game, 88 % of the subjects showed at least one significant change in all systemic variables; however, MBP and HR varied between subjects and epochs, while flux showed a consistent increase across all epochs. Group analysis of the systemic data for the fighting game across subjects shows a significant positive increase for MBP and HR for the second and third epochs and a significant positive increase for the flux signal across all epochs (Table 13.1). Group analysis of the systemic data for the puzzle game across subjects shows no significant increases for MBP and HR and a significant positive increase for the flux signal across all epochs (Table 13.1).

**Table 13.1:** Mean ( ± standard deviation) values for monitored physiological variables during three phases of the experiment. Statistically significant values are highlighted *p ≤ 0.05

	Puzzle			Fighting		
	1st epoch	2nd epoch	3rd epoch	1st epoch	2nd epoch	3rd epoch
**Δ[MBP](mmHg)**	3.7( ± 9.6)	5.3 ± (12.4)	5.3 ± (11.3)	0.3( ± 7.3)	7.4( ± 14.1)*	11.8( ± 23.5)*
**Δ[HR](beats/min)**	5.5( ± 19.8)	9.2 ± (22.5)	9.9 ± (25)	2.5( ± 5.9)	4.5( ± 8.1)*	9.9( ± 8.7)*
**Δ[Flux](no units)**	4.4( ± 5.5)*	7.9 ± (9.2)*	7 ± (9.1)*	7.5( ± 11.3)*	12.5( ± 19)*	11.4( ± 17)*

Correlation analysis revealed significant correlations between the HbO_2_ signal and systemic variables (Table 13.2). For the fighting game task, a significant correlation was seen at 40 % of channels between HbO_2_ and MBP, 42 % of channels between HbO_2_ and HR and 46 % of channels between HbO_2_ and flux. For the puzzle game task, a significant correlation was seen at 57 % of channels between HbO_2_ and MBP, 41 % of channels between HbO_2_ and HR and 70 % of channels between HbO_2_ and flux (Table 13.2).

**Table 13.2:** Correlation analysis with number representing the percentage of fNIRS channels

	MBP/HbO_2_	MBP/HHb	HR/HbO_2_	HR/HHb	Flux/HbO_2_	Flux/HHb
	**Puzzle game**					
***r*** **≥ 0.25**	57	26	41	22	70	26
**0.25 >** ***r*** **> − 0.25**	34	36	47	53	24	41
***r*** **≤ − 0.25**	9	38	12	25	6	33
	**Fighting game**					
r ≥ 0.25	40	20	42	14	46	9
**0.25 >** r **> − 0.25**	42	44	41	52	46	55
***r*** **≤ − 0.25**	18	36	17	34	8	36

## Discussion

We found significant localised changes in HbO_2_ and HHb measured over the frontal lobe during the gaming tasks. In addition, during the fighting game, our group analysis revealed significant changes in MBP and HR occurring at the middle and later periods of the activation block. We observed during both the fighting and puzzle games significant changes in the flux signal throughout the activation period. Correlation analysis between the HbO_2_ and HHb with the systemic measurements revealed some individual and channel variability with most of the HbO_2_ measurements correlating positively with flux in both the fighting and the puzzles game. There was a variation in the results among the volunteers between the two games. More haemodynamic activation patterns were seen during the puzzle game studies in the third epoch of the activation block compared to the other epochs, whereas in fighting game studies, these patterns were seen in the first period, just after the start of the activation block. In addition, during the fighting game studies, the percentage of activation patterns gradually decreases over time with the third epoch demonstrating the lowest percentage of volunteers showing activation.

Previous studies reported that playing videogames significantly increased systolic and diastolic pressure, heart rate and oxygen consumption in adolescents [8], in particular during the fighting game, which is a very dynamic game (the characters are moving, jumping and punching) that can possibly cause more areas in the brain to be activated (such as the motor cortex). The action involved in such a game can also cause emotional reactions in the subject. In a study exploring if playing a game that contains violence causes any sympathetic or parasympathetic reactions, the investigators concluded that a violent game causes different autonomic responses and affects heart rate and heart rate variability, which is a measure of stress reactivity [9]. In our studies, the high correlation coefficients found between fNIRS channels and systemic variables give an indication that some changes in fNIRS signals are due to changes in systemic variables and not due to haemodynamic changes originating from specific regions of the frontal lobe. Therefore, during analysis of brain activation during tasks such as video gaming, the contribution of the systemic changes should be taken into consideration.
